# A public resource of 15 genomically characterized representative strains of Shigella sonnei

**DOI:** 10.1099/mgen.0.001596

**Published:** 2026-01-12

**Authors:** Sydney L. Miles, Jane Hawkey, Ben Vezina, Vincenzo Torraca, Claire Jenkins, François-Xavier Weill, Stephen Baker, Kate S. Baker, Serge Mostowy, Kathryn E. Holt

**Affiliations:** 1 Department of Infection Biology, London School of Hygiene and Tropical Medicine, London, UK; 2 Department of Infectious Diseases, School of Translational Medicine, Monash University, Melbourne, Australia; 3 Gastrointestinal Bacterial Reference Unit, UK Health Security Agency, London, UK; 4 Institut Pasteur, Université Paris Cité, Unité des Bactéries pathogènes entériques, Centre National de Référence des Escherichia coli, Shigella et Salmonella, Paris, F-75015, France; 5 A*STAR Infectious Disease Labs, Biopolis, Singapore; 6 Department of Genetics, University of Cambridge, Cambridge, UK

**Keywords:** evolution, genomics, host adaptation, reference genomes, *Shigella sonnei*

## Abstract

*Shigella sonnei* is rapidly emerging as the dominant agent of shigellosis, an enteric disease responsible for a significant burden of morbidity and mortality worldwide. Whole-genome sequencing of *S. sonnei* isolated over the last three decades has revealed phylogenomic diversity within the population and the emergence of multiple lineages associated with distinct epidemiological patterns such as resistance to critical antimicrobials and/or transmission within different groups. However, most experimental work on *S. sonnei* biology and pathogenicity has focused on a single laboratory strain (53G), which is phylogenetically distant from currently circulating strains. Here, we introduce a set of phylogenetically diverse and epidemiologically relevant *S. sonnei* isolates made available through publicly accessible culture collections as a resource for laboratory science. We present their complete whole-genome sequences, including the pINV invasion plasmid (missing from a large proportion of public genome data due to loss during laboratory culture). Finally, the characterization and comparison of these complete genome sequences highlight evidence for ongoing adaptive evolution in *S. sonnei*, featuring the accumulation of insertion sequences, gene pseudogenization and structural variation.

**Significance as a BioResource to the community**

Genomic analysis of *Shigella* has historically been challenging due to the presence of hundreds of repetitive sequence elements (which can cause fragmented assemblies) and loss of the pINV invasion plasmid (essential to virulence) during laboratory culture. Furthermore, most experimental work on *Shigella sonnei* pathogenicity uses a lab strain that is phylogenetically distant from circulating isolates. To support *S. sonnei* experimental and *in silico* research and increase its relevance to current clinical problems, we report here the complete, high-quality genome sequences of 15 *S*. *sonnei* isolates, each selected to represent distinct sub-clades of epidemiological interest. We also make 13 of the corresponding strains publicly available in national reference culture collections.

## Data Summary

All sequencing reads and complete assemblies (with PGAP annotations) have been deposited into the National Center for Biotechnology Information (NCBI) database (accessions can be found in Table S1, available in the online Supplementary Material 1. Genome assemblies and Bakta annotations used in the analysis can be found in Figshare (https://doi.org/10.6084/m9.figshare.28302986) together with Mauve multiple-sequence alignments for the chromosome and pINV plasmid sequences and genome-scale metabolic models produced for each strain.

Pure cultures of 13 strains were deposited in the publicly accessible National Collection of Type Cultures (NCTC, UK) or the ‘*Collection de l’Institut Pasteur*’ (CIP, France) (accessions listed in Table S1).

## Introduction

*Shigella* are human-adapted lineages of *Escherichia coli* which have evolved to cause severe diarrhoeal disease, known as shigellosis. Shigellosis is the second leading cause of diarrhoeal deaths globally, most of which occur in children under 5 years of age in low- and middle-income countries [1].

Within *Shigella*, there are four recognized subgroups that are separated based on their antigenic properties: *Shigella boydii*, *Shigella dysenteriae*, *Shigella flexneri *and *Shigella sonnei*. Each *Shigella* subgroup emerged from *E. coli* following the acquisition of a large virulence plasmid (pINV, 210–240 kbp) which conferred the ability to invade human cells [2,3]. Aside from pINV acquisition, several other gain- and loss-of-function events have been established as stepwise evolutionary changes which facilitated the human adaptation of *Shigella* [4,5]. Throughout the course of pathoadaptation, considerable gene loss has been observed [5], a process that is also documented in other host-restricted pathogens such as *Salmonella enterica* serovar Typhi, *Bordetella pertussis* and *Mycobacterium leprae* [6,8]. In the case of *Shigella*, gene loss is mostly associated with insertion sequences (ISs), small transposable elements that can mobilize within the genome, disrupt coding sequences and mediate genome rearrangements, insertions and deletions [5,9]. Interrogation of *Shigella* and *E. coli* genomes has revealed that each *Shigella* subgroup harbours significantly more copies of ISs than other *E. coli* pathotypes [5,10].

*S. sonnei* represents the youngest and least genetically diverse *Shigella* subgroup [11], with multidrug-resistant clones emerging as the dominant agent of shigellosis in both high-income and economically transitioning countries [12,14]. A hierarchical single nucleotide variant (SNV)-based framework has been applied to genotype *S. sonnei* which sees the current population of *S. sonnei* delineated into five main lineages (separated by 600 pairwise SNVs), each with varying degrees of global dissemination and expansion [15]. Lineage 1 represents an ancestral lineage rarely detected outside of Europe; lineage 4 represents an extinct lineage comprising a single known isolate [11]; lineage 5 is restricted to Latin America and parts of Africa [16]. Lineage 2 has undergone limited dissemination, establishing localized clones in some regions, but overall, lineage 3 has been the most successful at global dissemination having been detected on every continent and today represents the most epidemiologically significant lineage [15]. Within lineage 3, clades 3.6 (Central Asia III/CipR) and 3.7 (Global III) dominate the epidemiological landscape, with regional studies highlighting a pattern of clonal replacement and expansion, seemingly driven by the independent acquisitions of genes conferring multidrug resistance [encoded on a class II integron (Int*2*)-bearing transposon, Tn*7*] [13,17].

Increased resistance to antimicrobials is a clear signature of lineage 3 *S*. *sonnei*, but there is evidence of ongoing adaptation elsewhere in the genome, marked by the expansion of ISs and the loss of catabolic genes [10,11]. However, a lack of completed genomes (and even fewer genomes containing pINV [11], which is readily lost during laboratory culture due to the deletion of toxin-antitoxin systems involved in plasmid maintenance [18]) has left gaps in our knowledge of IS-mediated diversity and genome variability. Furthermore, genomic data reveals that the classical laboratory strain 53G (isolated in 1954 and used for the vast majority of experimental work on *S. sonnei* [19]) belongs to lineage 2 which is now comparatively rare amongst clinical isolates and is quite distant from the dominant lineage 3 [11,15].

Here, we present the complete genomes of 15 pINV-containing *S. sonnei* isolates, including representatives from lineages 1, 2 and 3. The comparison of completed genomes supports ongoing adaptive evolution in lineage 3, characterized by increased IS abundance, gene pseudogenization and genomic rearrangements. Overall, the results presented here provide novel insights into the genomic variation of *S. sonnei* lineages and significantly further the resources available for the study of *S. sonnei*.

## Methods

### Bacterial strains used in this study

*S. sonnei* isolates used in this study are listed in Tables 1 and S1. Isolates were originally collected as part of routine public health surveillance in the UK [20] and France [21], or as part of a cohort study in Vietnam [22]. Bacterial isolates were fully anonymized and de-identified prior to their use in this study. Strains were received on agar slants or stabs and streaked onto tryptic soy agar plates, supplemented with 0.01% Congo Red to select for pINV+ isolates [23]. Smooth, red colonies (which indicate T3SS and pINV presence) were picked and stored in 25% (v/v) glycerol at −80 °C.

**Table 1.** Summary of *S. sonnei* genome assemblies presented here

Strain ID	Genotype*	Genome size (bp)	ONT depth	CDS†	tRNA	Contig	Chromosome size (bp)	pINV size (bp)	Other plasmids
201809330	1	5,389,660	61×	5428	97	7	4,916,714	242,731	5
391324	1.5	5,260,824	59×	5255	97	4	4,888,493	238,313	2
356538	2.1	5,170,588	76×	5176	97	4	4,946,399	216,858	2
830292	2.3	5,150,027	60×	5166	96	3	4,924,717	220,157	1
373220	2.12.4	5,289,501	47×	5306	93	4	4,983,563	223,662	2
590907	3.4.1	5,174,681	74×	5229	97	7	4,940,164	215,149	5
623218	3.6.1	5,112,917	68×	5154	97	9	4,862,817	214,316	7
02_1157	3.6.1.1.1	5,073,758	86×	5110	96	5	4,832,017	212,976	3
642321	3.6.2	5,100,173	43×	5135	97	6	4,866,958	213,229	4
633497	3.7.11	4,986,415	46×	5046	93	7	4,657,310	214,646	5
598955	3.7.16	5,039,937	54×	5067	98	7	4,807,305	214,536	5
618335	3.7.28	5,138,354	61×	5177	98	6	4,806,102	214,666	4
03_0142	3.7.29.1.4	5,156,461	86×	5182	96	9	4,824,935	214,579	7
627346	3.7.30.1	5,122,274	42×	5168	100	6	4,884,953	216,152	4
381259	3.7.30.4.1	5,260,132	58×	5322	91	8	4,933,886	214,700	6
53G‡	2.8	5,220,473	na	5248	96	5	4,988,504	215,774	3

*Genotypes were predicted using the *S. sonnei *genotyping framework implemented in Mykrobe.

†CDS and tRNA were predicted using Bakta.

‡Features of the completed genome of the classical laboratory strain, 53G, are included for comparative purposes.

CDS, coding sequence; ONT, Oxford Nanopore Technologies.

### Genome sequencing

The DNA extraction and long-read (Oxford Nanopore Technology) and short-read (Illumina) sequencing of *S. sonnei* clinical isolates were performed by MicrobesNG as a paid service. Briefly, an overnight culture was grown in tryptic soy broth (TSB) (incubation at 37 °C, 400 r.p.m.) and sub-cultured to prepare isolates for sequencing. Once the sub-culture reached mid-exponential phase, cells were pelleted by centrifugation at 4,000 ***g*** for 4 min, washed in 1 ml PBS and resuspended at a density of 4×10^9^ cells in 500 µl DNA shield (Zymo Research). For Illumina sequencing, paired-end libraries were prepared with the Nextera XT Library Prep Kit and sequenced on an Illumina NovaSeq 6000 instrument to generate 2×250 bp paired-end reads (472.50–811.06 Mbp per isolate). For ONT sequencing, libraries were prepared with the SQK-RBK114.96 kit (Oxford Nanopore Technologies) and barcoded samples were pooled together into a sequencing library. The library was loaded onto an R.10.4.1 flow cell and sequenced over seven runs (until the desired coverage was reached) on a GridION instrument. Across the seven runs, run time ranged from 4 to >24 h and 262.25–397.69 Mbp of data per isolate was generated. Reads were basecalled using Dorado (model r1041_e82_400bps_hac_v4.2.0).

### *De novo* genome assembly

Prior to genomic analysis, sequencing reads were quality checked using FastQC (v.0.12.0) [24], trimmed using Filtlong (v.0.2.1) [25] (for long reads) or Trimmomatic (v.0.4.0) [26] (for short reads). Genomes were then assembled using the Hybracter (v.0.7.3) [27] long-read first assembly pipeline, with Flye (v.2.9.4) [28] selected as the long-read assembler. As part of the pipeline, genomes were polished with Medaka (v.1.8.0) first and then with short reads using PyPOLCA (v.0.3.1) [29,30]. The quality of assemblies was checked using Quast (v.5.0.2) [31] [using 53G as a reference genome (accession NC_016822)]. Completeness and contamination were analysed using CheckM (v.1.2.3) [32]. Contigs were concatenated into a multifasta file using SeqKit (v.2.8.2) [33], and then genomes were annotated using Bakta (v.1.9.3) [34], with the full database option selected.

### Genome characterization

Complete genomes were genotyped using the *S. sonnei* genotyping framework implemented in Mykrobe (v.0.13.0) [15,35], to confirm *S. sonnei* lineage assignments matched those obtained previously from published Illumina sequence data for the same isolates [15]. Plasmid sequences were replicon-typed using MOB-suite (v.3.1.9) [36] ‘Mob-typer’ function. Antimicrobial resistance (AMR) determinants were identified using NCBI AMRFinderplus (v.3.12.8) [37]. Colicins were identified using ABRicate (v.1.0.1) [38] using the custom colicin database created by De Silva *et al*. [39] (https://figshare.com/articles/dataset/colicin_database/20768260/1?file=37009930).

IS elements were identified using ISEScan (v.1.7.2.3) [40], and results were filtered to identify ISs present in the chromosome, pINV and other plasmids. Bakta did not efficiently annotate pseudogenes (tested on 53G for which the number of pseudogenes was previously reported [5,10]), so the prokaryotic genome annotation pipeline (PGAP) (v.6.7) [41] was used to quantify and compare pseudogenes.

### Pangenome analysis

Pangenome analysis was performed using Panaroo (v.1.5.0) [42] using the ‘strict’ mode, MAFFT (v.7.526) [43] was selected to perform core genome alignment and otherwise default settings were used. The phylogenetic tree for pangenome visualization was produced using the core genome alignment file generated by Panaroo as an input to FastTree (v.2.1.1) [44] which was run using the generalized time-reversible model and otherwise default settings. Visualization was performed using Phandango (accessed on 18 August 2024) [45]. Unique homologous gene clusters (HGCs) for each lineage were classified into biological process Gene Ontology (GO) categories using the PANTHER functional classification system (v.19.0) [46].

### Whole-genome alignment

Complete genome sequences were reoriented to the *fabB* gene where necessary due to an inverted collinear block involving the *dnaA* gene. Whole-genome alignments were performed using progressive Mauve (v.2.4.0) [47]. This approach generates locally collinear blocks which enable the visualization of structural genome rearrangements. Alignments were manually inspected for any rearrangements (inversions, deletions or insertions) that were greater than 7 kb. The structural variants were confirmed by mapping the long reads back to the assembly and checking for congruence. To create isolated gene cluster comparison figures, relevant gene clusters were extracted following visualization in Mauve and aligned using Clinker (v.0.0.29) [48].

### Genome-scale metabolic models

Metabolic models were constructed using CarveMe (v1.5.1) with the following options: ‘-u gramneg -g M9’ [49]. Metabolic reaction presence/absence was generated using reaction_presence_absense_generate.py (https://github.com/bananabenana/Metabolic_modelling_scripts/tree/main/reaction_pres_abs). Growth simulations were then performed with Bactabolize (v1.0.3) using the ‘fba’ command [50] whereby each metabolite with an exchange reaction was tested as a sole carbon, nitrogen, phosphorus and sulphur nutrient source.

## Results and discussion

### The complete genomes of 15 phylogenetically diverse *S. sonnei* isolates

*S. sonnei* isolates were selected based on existing short-read (Illumina) data to represent the diversity of the current *S. sonnei* population (for which lineage-associated epidemiological trends are detailed in Table 2 and Fig. 3 of Hawkey *et al*. [15]). They comprised two lineage 1 isolates, four lineage 2 isolates (including 53G) and ten lineage 3 isolates (Table S1). We initially sought to include representatives from lineages 4 and 5 but found that cultures were either non-viable following many years of laboratory storage or simply had lost pINV (including the only known lineage 4 isolate) and thus were unsuitable for inclusion. Complete chromosomal and pINV sequences were obtained using hybrid short- and long-read sequencing; general features of the newly sequenced isolates are summarized in Table 1.

**Table 2.** Summary of AMR determinants identified by AMRfinderplus Plasmid-encoded determinants are displayed in black, and chromosomally encoded determinants are shown in blue. The column titled ‘AMR encoding plasmids’ indicates the type (as indicated by MOB-suite), sizes and accessions of plasmids encoding AMR determinants in each strain. na, not applicable; no AMR encoding plasmids identified in this strain.

Antibiotic class										
Genotype	Strain ID	Beta-lactam	Aminoglycoside	Trimethoprim	Sulphonamide	Phenicol	Tetracycline	Streptothricin	Quinolone	AMR encoding plasmids
1	201809330	blaTEM-1	aph(6)-Id, aph(3″)-Ib	dfrA14	sul2					Inc1, 102,906 bp IncI2, 65,348 bp IncI2, 43,917 bp
1.5	391324									na
2.1	356538	blaOXA-1	aadA1			catA1	tet(B)			na
2.3	830292									na
2.8	53G									na
2.12.4	373220	blaTEM-1	aadA1, aph(6)-Id, aph(3″)-Ib	dfrA1	sul2		tet(B)	sat2		IncFIA/IncFII, 77,123 bp
3.4.1	590907	blaOXA-1	aadA1			catA1	tet(B)		qnrB19	Non-typable, 2,699 bp
3.6.1	623218		aph(6)-Id, aph(3″)-Ib	dfrA1	sul2		tet(A)	sat2	gyrA_S83L	Non-typable, 8,401 bp
3.6.1.1.1	02_1157		aph(6)-Id, aph(3″)-Ib	dfrA1	sul2		tet(A)	sat2	gyrA_S83L, gyrA_D87G, parC_S80I	Non-typable, 8,401 bp
3.6.2	642321		aph(6)-Id, aph(3″)-Ib	dfrA1	sul2		tet(A)	sat2		Non-typable, 10,093 bp
3.7.11	633497		aadA1	dfrA1				sat2		na
3.7.16	598955		aadA1	dfrA1				sat2		na
3.7.28	618335		aadA1	dfrA1, dfrA51				sat2		na
3.7.29.1.4	03_0142		aadA1, aph(6)-Id, aph(3″)-Ib	dfrA1	sul2			sat2	gyrA_S83L	na
3.7.30.1	627346		aadA1	dfrA1				sat2		na
3.7.30.4.1	381259		aadA1	dfrA1				sat2		na

There was some variation in genome size, with chromosome sizes ranging from 4.65 to 4.98 Mbp. In line with expectations from prior reports on genome reduction [10], lineage 1 genomes had an average chromosome size of 4.90 Mbp (range 4.89 to 4.92 Mbp), lineage 2 chromosomes averaged at 4.96 Mbp (range 4.92 to 4.99 Mbp), whilst lineage 3 chromosomes were the smallest, averaging at 4.84 Mbp (range 4.66 to 4.94 Mbp). Likewise, pINV sizes ranged from 212 to 242 kbp, and pINV in lineage 1 genomes was ~24 kbp larger than lineage 2 and 3 genomes (average 240 vs 216 kbp).

**Fig. 1.**
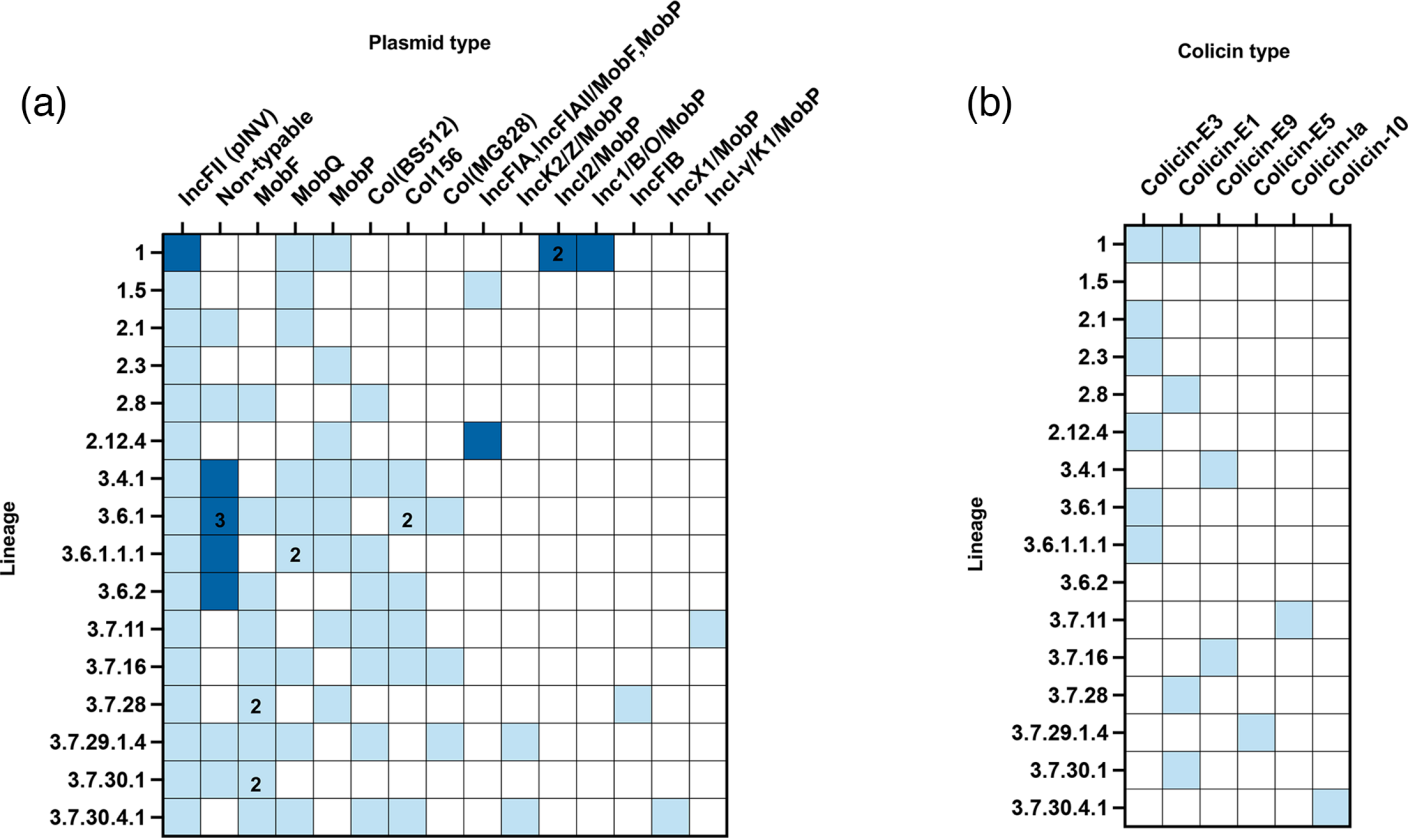
Summary of plasmid content and colicin presence in *S. sonnei* assemblies presented here. (**a**) Plasmids were typed using the MobTyper function in MOB-suite. White squares indicate the absence of the plasmid type, light blue indicates its presence and dark blue indicates that the plasmid type is present and encodes AMR determinants. Where a number is printed in the cell, this indicates multiple of the same plasmid type present in a single strain. (**b**) Colicins were identified using ABRicate and the full colicin database created previously [39]. White indicates the absence of colicin type, and light blue indicates its presence.

In addition to the chromosome and pINV, each genome also harboured additional plasmids (between 1 and 8 per genome, ranging from 1,459 to 108,503 bp) (Fig. 1a, Table S2). Plasmid typing using MOB-suite revealed a diverse plasmid repertoire, with Col plasmids (colicinogenic plasmids which encode the genes required for colicin production [51]) being frequently detected. Colicins are bactericidal proteins (bacteriocins) which are known to drive the antibacterial activity of *S. sonnei* and are thought to aid in competition within the intestinal niche [39,52]. The carriage of colicins E3 (which arrests protein synthesis [53]) and E1 (which depolarizes the cell membrane [54]) was most common (Fig. 1b, Table S3) and has been associated with local lineage replacement in *S. sonnei* [13,39], although no global lineage-associated trends were evident amongst the selected isolates.

### Carriage of AMR determinants is representative of known lineage associations

Screening assemblies for known AMR determinants confirmed that the isolates we selected were representative of the AMR profiles that had previously been associated with the genotypes they were selected to represent (detailed in Fig. 3 of Hawkey *et al*. [15]). Isolates belonging to clade 3.6.1 were both found to carry the GyrA-S83L mutation associated with this sub-clade [55]; the isolate representing subclade 3.6.1.1.1 also carried the additional mutations GyrA-D87G and ParC-S80I, which are characteristic of subclade 3.6.1.1 and its descendants, and are responsible for its high-level fluoroquinolone resistance [56]. All clade 3.6 isolates carried Tn*7* harbouring *dfrA1* and *sat2* in the integron cassette (conferring resistance to trimethoprim and streptothricin), which was located between an IS*4*-family transposase and *glmS*. Similarly, all clade 3.7 isolates carried a distinct Tn*7* variant at the same chromosomal locus (Fig. S1A) harbouring *dfrA1*, *sat2* and *aadA1* (conferring aminoglycoside resistance). Additionally, all isolates belonging to clade 3.6 were found to carry the small spA plasmid, encoding *tetA*, *aph(6)-Id, aph(3″)-Ib* and *sul2* (Tables 2 and S4), as is commonplace within this clade [55]. Genotypes 2.1 and 3.4.1 were both found to harbour distinct variants of the chromosomally located *Shigella* resistance locus (SRL) which encodes for *aadA1*, *tetB*, *catA1* and *bla*_OXA-1_, typical of Latin American-associated *S. sonnei* (Fig. S1B) [57]. Insertions of the SRL occurred at different sites in the chromosome, but in both cases, they were inserted into a copy of *trnS* (which encodes for tRNA-Ser), consistent with previous reports [58]. Until now, the location of some resistance determinants as either chromosomally encoded (and so stably fixed) or plasmid encoded (which may be more easily lost) was unclear due to a lack of completed genomes. Resolution of the location of resistance determinants may be beneficial for future use in experimental work, where stable chromosomally encoded genes may act as selective markers.

### Pangenome analysis highlights limited lineage-associated gene content variation

**Fig. 2.**
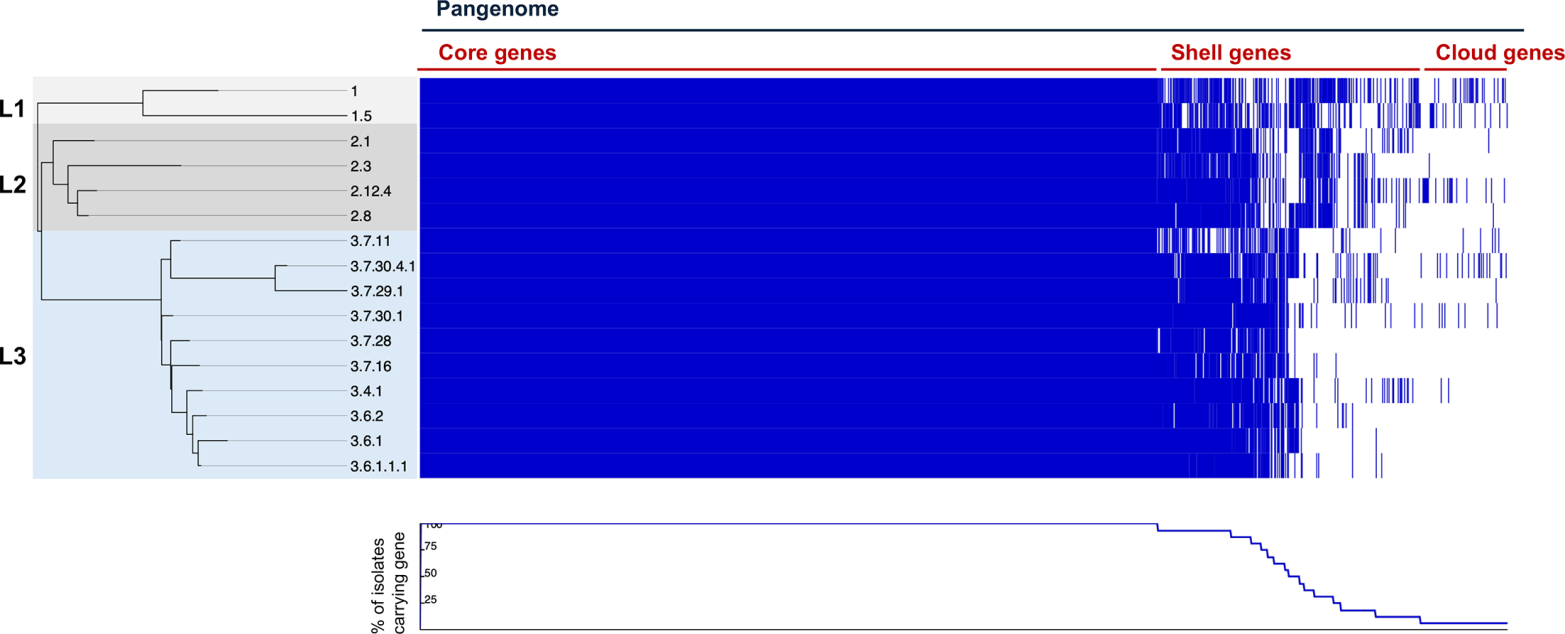
Linear visualization of the *S. sonnei* pangenome plotted alongside a maximum-likelihood phylogenetic tree. Panaroo was used to build a pangenome and a core genome alignment. FastTree was used to construct a maximum likelihood tree, which is rooted at the midpoint. The resulting outputs were then visualized using Phandango. Blue stripes indicate the presence of a gene, and white stripes indicate the absence of a gene. Core genes are those present in all genomes; shell genes are defined by the presence in ≥3 genomes; cloud genes are those present in <3 genomes.

To explore overall gene content variation within *S. sonnei* lineages, a pangenome analysis was performed using Panaroo, including the 15 novel isolates and the laboratory strain 53G. The resulting pangenome was found to be open (*γ*=0.07) and a total of 6,317 HGCs were identified: of these, 4,282 genes were present in all 16 genomes, representing the core genome; 1,268 genes were present in ≥3 strains, representing ‘shell’ genes and 767 genes were present in <3 strains representing the ‘cloud’ genes (Fig. 2). The number of unique HGCs varied from 1 to 104 per isolate (Table 3). Lineage 1 had the most lineage-specific HGCs (HGCs present in every isolate of that lineage and absent in the other lineages) at 63, consistent with previous reports of gene loss in lineages 2 and 3 compared to lineage 1 [10]. Lineage 2 had only 6 lineage-specific HGCs and Lineage 3 had 27 lineage-specific HGCs. Many lineage-specific HGCs in lineages 2 and 3 were annotated as ISs or IS accessory genes (discussed below). These results highlight minimal fixed gene content variation between *S. sonnei* lineages, suggesting that variation in the success of lineages is not largely driven by gene content alone.

**Table 3.** Number of HGCs unique to each isolate and to each lineage. Unique genes were annotated using Bakta and HGCs were identified using Panaroo

Lineage	No. of genes unique to isolate	No. of genes unique to lineage
1	95	63
1.5	104	
2.1	9	6
2.3	18	
2.8	6	
2.12.4	78	
3.4.1	24	27
3.6.1	5	
3.6.1.1.1	3	
3.6.2	3	
3.7.11	3	
3.7.16	3	
3.7.28	1	
3.7.29.1.4	11	
3.7.30.1	31	
3.7.30.4.1	90	

### Variations in the carriage of ISs

**Fig. 3.**
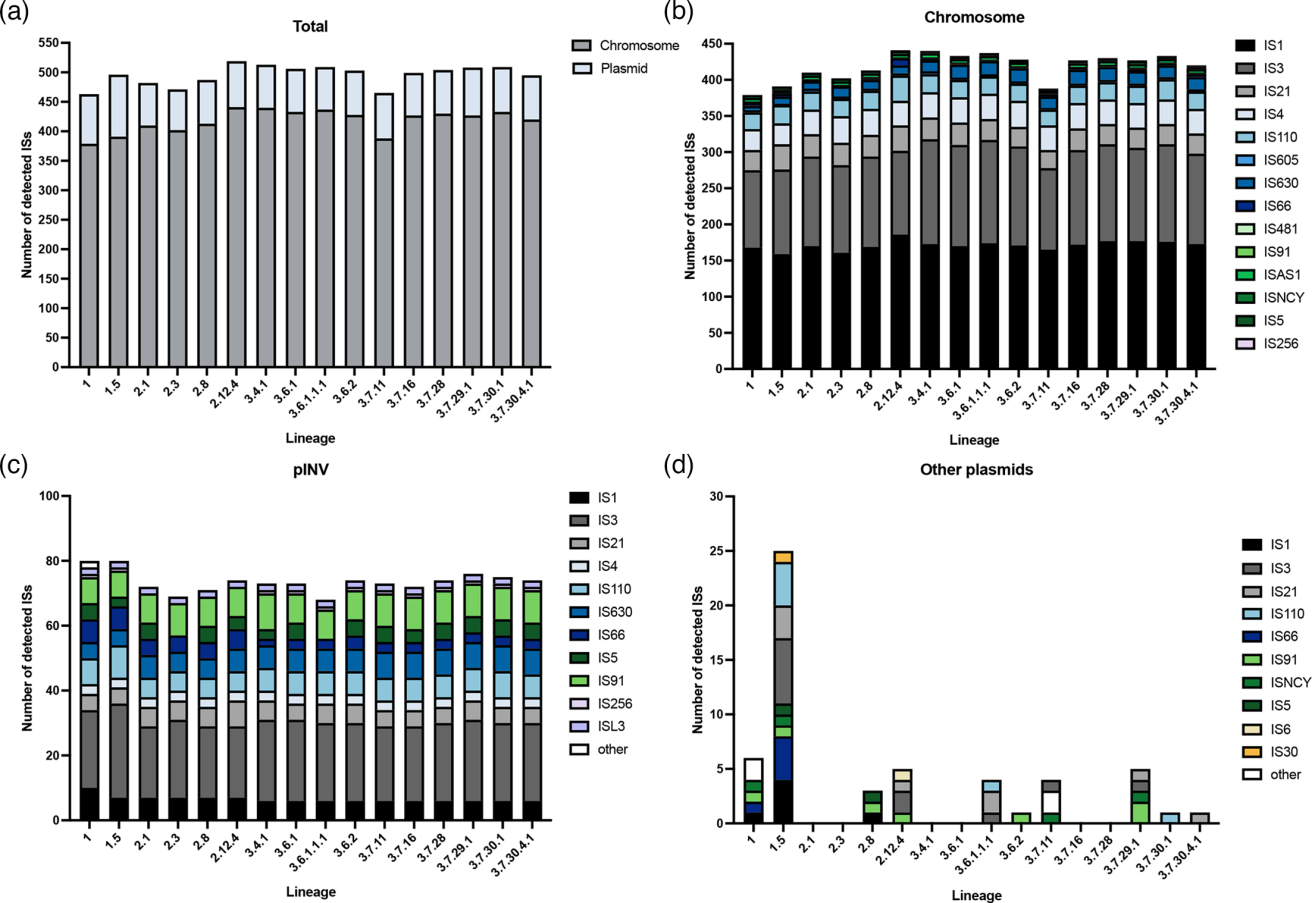
ISs detected in complete *S. sonnei* genome sequences by ISEscanner. (**a**) Total number of ISs identified across all contigs of completed genomes and their location. (**b**) ISs identified in completed chromosomal sequences. (**c**) ISs identified in pINV sequences. (d) ISs identified in other plasmids present in *S. sonnei* genomes. ISs were detected using ISEscanner, ‘other’ represents ISs that could not be matched to a known IS family within the database used.

Previous studies have produced evidence for ongoing IS activity within *S. sonnei*, with data from short-read sequences highlighting the proliferation of ISs specifically within lineages 2 and 3 [10]. However, this has not been examined in completed chromosomal sequences, and never in pINV. We therefore examined the abundance and composition of ISs across *S. sonnei* lineages using our reference genomes. A total of 16 distinct IS families were identified across all genomes (Fig. 3a). There was some variation in the total IS burden by lineage, with lineage 1 genomes harbouring the fewest, with an average of 480; lineage 2 had an average of 490, whilst lineage 3 genomes had an average of 501.

The detected ISs were split based on their presence in the chromosome, pINV or other plasmids to account for their different evolutionary histories and dynamics. Considering the abundance of ISs in the chromosome alone revealed a clearer trend, with lineage 1 harbouring the fewest chromosomal ISs (median 385, range 379 to 391) and lineage 3 harbouring the most (median 429, range 388 to 440) (Fig. 3b). In pINV, a total of 12 different IS families were identified and two were unique to pINV: IS*5* and IS*L3*, neither of which has been reported in *S. sonnei* before to our knowledge, likely owing to a scarcity of pINV sequences. A contradictory trend in IS abundance in pINV (compared to chromosomal IS abundance) was observed (Fig. 3c), consistent with pINV being larger in lineage 1, although the differences in IS count were smaller compared to chromosomal ISs (median 80 in lineage 1, median 71.5 in lineage 2 and median 73.5 in lineage 3). For ISs carried on other plasmids, lineage 1.5 harboured the most (*n*=25), which is consistent with its carriage of the largest plasmid (126 kbp IncFIA, IncFII/MOB_F_, MOB_P_ plasmid, accession: CP179999; Fig. 3d). ISs carried on other plasmids were sporadically detected in single strains, but there was no clear lineage-associated pattern, in line with the limited lineage-associated trends in plasmid carriage.

### Lineage 3 genomes harbour more pseudogenes

The accumulation and proliferation of ISs have been linked to increased formation of pseudogenes (inactivated gene remnants) [59]. To investigate any changes in gene functionality, genomes were screened for the presence of pseudogenes using PGAP. The number of pseudogenes predicted using this method is higher than previously published predictions (for 53G at least [10]), but this is likely due to differences in the annotation method, whereby PGAP may be recording 2 separate pseudogenes in situations where a single gene is interrupted by an IS thus creating two fragments.

**Fig. 4.**
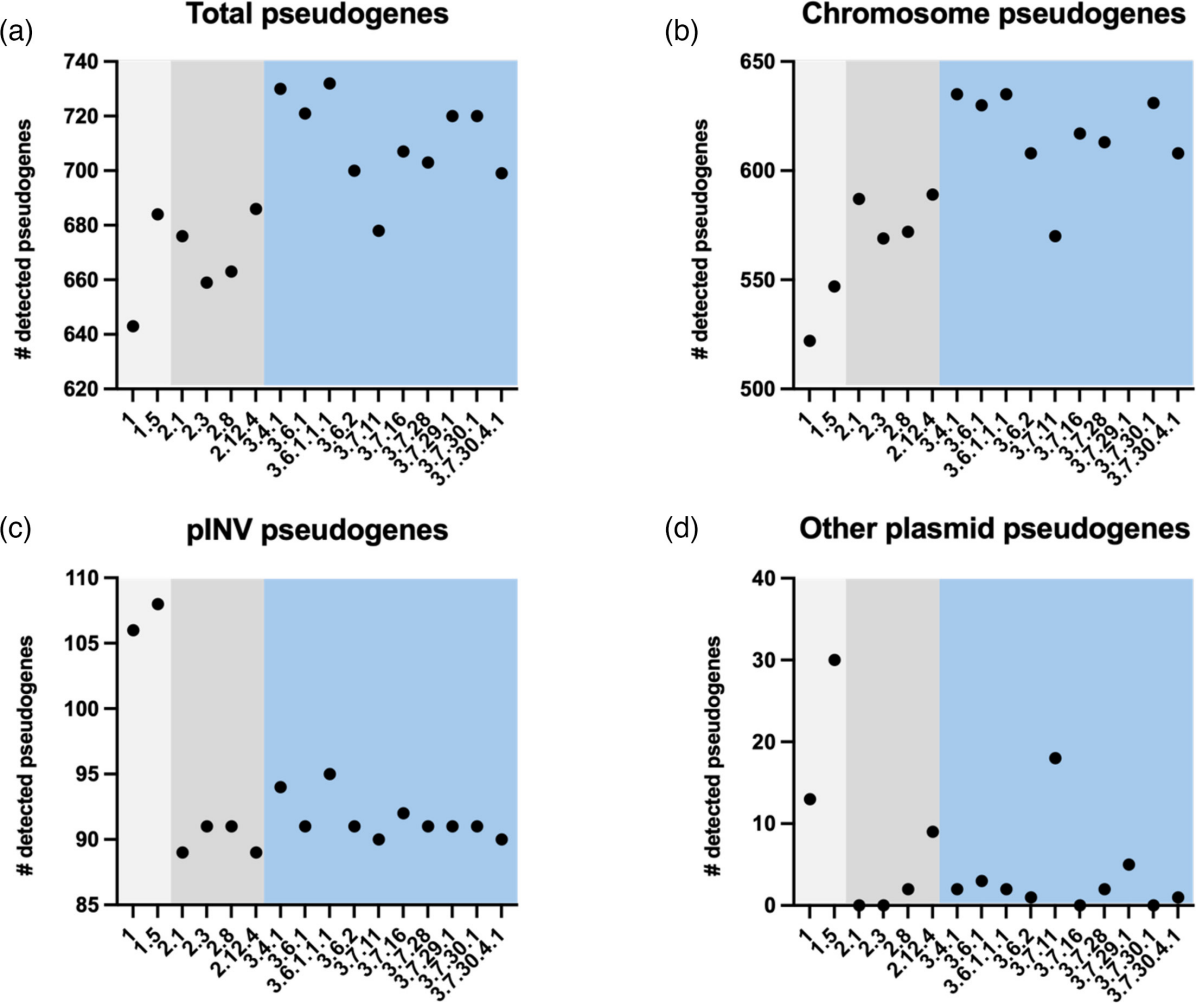
Abundance of pseudogenes predicted by the NCBI prokaryotic genome annotation pipeline (PGAP). (**a**) Total pseudogenes identified across all contigs of completed genomes. (**b**) Pseudogenes identified in completed chromosomal sequences. (**c**) Pseudogenes identified in pINV sequences. (**d**) Pseudogenes identified in other plasmids present in *S. sonnei* genomes. Lineage 1 isolates are in light grey, lineage 2 in dark grey and lineage 3 in blue.

In agreement with the increased abundance of ISs in lineage three isolates, this analysis also revealed a similar increase in the total number of pseudogenes present in lineage 3 genomes, with mean 711 identified compared to 671 in lineage 2 and 663.5 in lineage 1 (Fig. 4a–d). The differences in pseudogene number were mostly concentrated in the chromosome, where the lineage 3 chromosomes harboured the most pseudogenes (Fig. 4b). In pINV, the same pattern of IS abundance was observed in the number of pseudogenes, where lineage 1 genomes had the greatest abundance of pseudogenes (Fig. 4c), likely due to the larger pINV size.

### Structural variation in *S. sonnei* genomes

*Shigella* genomes are documented to have an exceptionally high rate of structural variation compared to other pathogenic (and non-pathogenic) *E. coli* [60], likely mediated by the large complement of ISs in their genomes [10]. To investigate lineage-associated structural variation, whole-genome alignments of chromosomal and pINV sequences were inspected (represented in Figs S2 and S3).

In the chromosomes, we identified four large-scale (>7 kbp) variations that were conserved within lineages (summarized in Table 4), two of which were identified in all lineage 2 and 3 genomes, and two of which occurred in only lineage 3 genomes. In lineages 2 and 3, of particular note was the absence of *xylFGH* and *xylR*, which have a role in xylose utilization [61]*,* which suggests adaptive loss of metabolic function, since these genes are also disrupted in other *Shigella* subgroups [5]. Another structural variant included the absence of the type 1 fimbrial operon in all lineage 3 genomes and distinct partial disruptions of the operon in lineage 1 and 2 genomes, as described fully in [62]. In agreement, recent work described the specific loss of putative immunogenic components (including type 1, K88 and Curli fimbrial loci), whilst other determinants of pathogenicity (including the T3SS and O-antigen encoding regions) remained highly conserved [62]. Furthermore, the convergent disruption of type 1 fimbriae has been previously reported in all other *Shigella* subgroups [63], suggesting that its presence is detrimental to *Shigella*, and thus, its parallel loss in multiple lineages may reflect adaptive evolution.

**Table 4.** Summary of conserved, large-scale structural variants identified Only variants conserved in every isolate of each lineage and those over 7 kbp are reported here.

Type	Description	Gene involved	IS flanked?
Deletion	~22 kbp absent in all lineage 2 and 3 genomes	xylFGH, xylR, bax, baxL, malS, avtA, ES036, ysaA, yiaJKLMNO, lyxK, ulaD, sgbU, araD, yesN, melB	No
Deletion	~7 kbp absent in all lineage 2 and 3 genomes	ISSd1, IS3, ISEc27, IS630, hypothetical protein, yehM, yehL, zorO	No
Deletion	~16 kbp absent in all lineage 3 genomes	yahBCD, pdeL, ehaA, hypothetical protein, betT, betI, betAB, unnamed (locus tag IAHEGK_19900)	Flanked by IS3 family transposase on one side
Deletion	~7 kbp absent in all lineage 3 genomes	Hypothetical protein, hypothetical protein, fimHGFDB, ISSso6	No
Inversion	~20 kbp inversion in all lineage 2 and 3 genomes	idnO, idnT, idnR, yjgR, lptGF, pepA, holC, valS, yjgN, istB, IS21, IS1, yjgM, rraB, argF, hypothetical protein, hypothetical protein, hypothetical protein	No
Inversion	~45 kbp inversion in all lineage 2 genomes, ~20 kbp inversion in all lineage 3 genomes	Lineage 2: raiA, hypothetical protein, pheA, tyrA, aroF, hypothetical protein, yfiR, ISSd1, ISSfl10, hypothetical protein, IS21, istB, dgcN, yfiB, rplS, trmD, rimM, rpsP, ffh, ypjD, grpE, nadK, recN, bamE, IS4, yfjF, ratA, smpB, ssrA, 16 unnamed phage related proteins, gpL, ISSd1, ISSfl10, unnamed phage protein Lineage 3:, yfjF, ratA, smpB, ssrA, 16 unnamed phage related proteins, gpL, ISSd1, ISSfl10, unnamed phage protein	IS4 family transposase flanked
Inversion	~40 kbp inversion in all lineage 3 genomes	Hypothetical protein, hypothetical protein, ansB, yggN, yggL, trmB, mutY, yggX, mltC, nupC, hypothetical protein, IS110, ldcC, yqgH, yqgA, trnF, hypothetical protein, ISEc11, hypothetical protein, dNA2, hypothetical protein, sigA, hypothetical protein, yghQ, yghR, yghS, yghR	Flanked by IS630 on one side
Inversion	~34 kbp inversion in all lineage 3 genomes	IS2, IS2, araA1, rclC, rclA, rclR, ykgEFG, hypothetical protein, hypothetical protein, yahEFG, yahIJK, hypothetical protein, yahL, IS3, ISSfl10, yahN, hypothetical protein, hypothetical protein, lacZ, lacI, hypothetical protein, mhpAB, hypothetical protein, IS600	IS3 family transposase flanked
Inversion	~130 kbp inversion in clades 3.4 and 3.6, ~107 kbp translocation inversion in clade 3.7	Listed in supplementary material	No
Deletion*	~11 kbp deletion in all lineage 3 genomes and lineage 1.5	Rhs element protein, faeG, faeDEF, cshE, faeH, faeI, ybdN, spo0j	Flanked by IS66 on one side

*Structural variation detected on pINV; all other variations were found to be chromosomal.

We identified five large-scale inversions conserved within lineages which were confirmed by mapping long-reads to the complete assemblies. Two were identified in all lineage 2 and 3 genomes (~20 and 45 kbp), and in this case, both were flanked by IS*4*-family transposases, suggestive of IS-mediated homologous recombination. Three inverted regions were identified uniquely in lineage 3 genomes, but only one of these was flanked by IS copies, suggesting that other mechanisms may also be driving structural variation in *S. sonnei*. The implications of these chromosomal rearrangements remain ambiguous, but potential impacts on gene expression, or inactivation, are likely important for conferring adaptive changes. Furthermore, lineage-associated variations identified here might indicate differential niche occupation, since rearrangements are known to contribute to bacterial adaptability [64].

For pINV, we observed limited structural variation, and the differences in pINV size (described above) did not seem to be mediated by a single large structural rearrangement, but instead by smaller-scale deletions. The most prominent example is an ~11 kbp loss shared by all lineage 3 genomes in our reference set, as well as lineage 1.5, which included the region encoding for a K88 fimbriae (previously reported to play a role in the pathogenesis of enterotoxigenic *E. coli* [65] and recently described as absent from lineage 3 *S*. *sonnei* [62]). The remaining difference can largely be attributed to the increased abundance of ISs within lineage 1 pINV, which contains an additional 9–14 kbp of IS content. The overall lack of variation observed here is consistent with an essential role for pINV maintenance in the pathogenesis and spread of *S. sonnei* [66].

### Metabolic capacity of *S. sonnei* genotypes

**Fig. 5.**
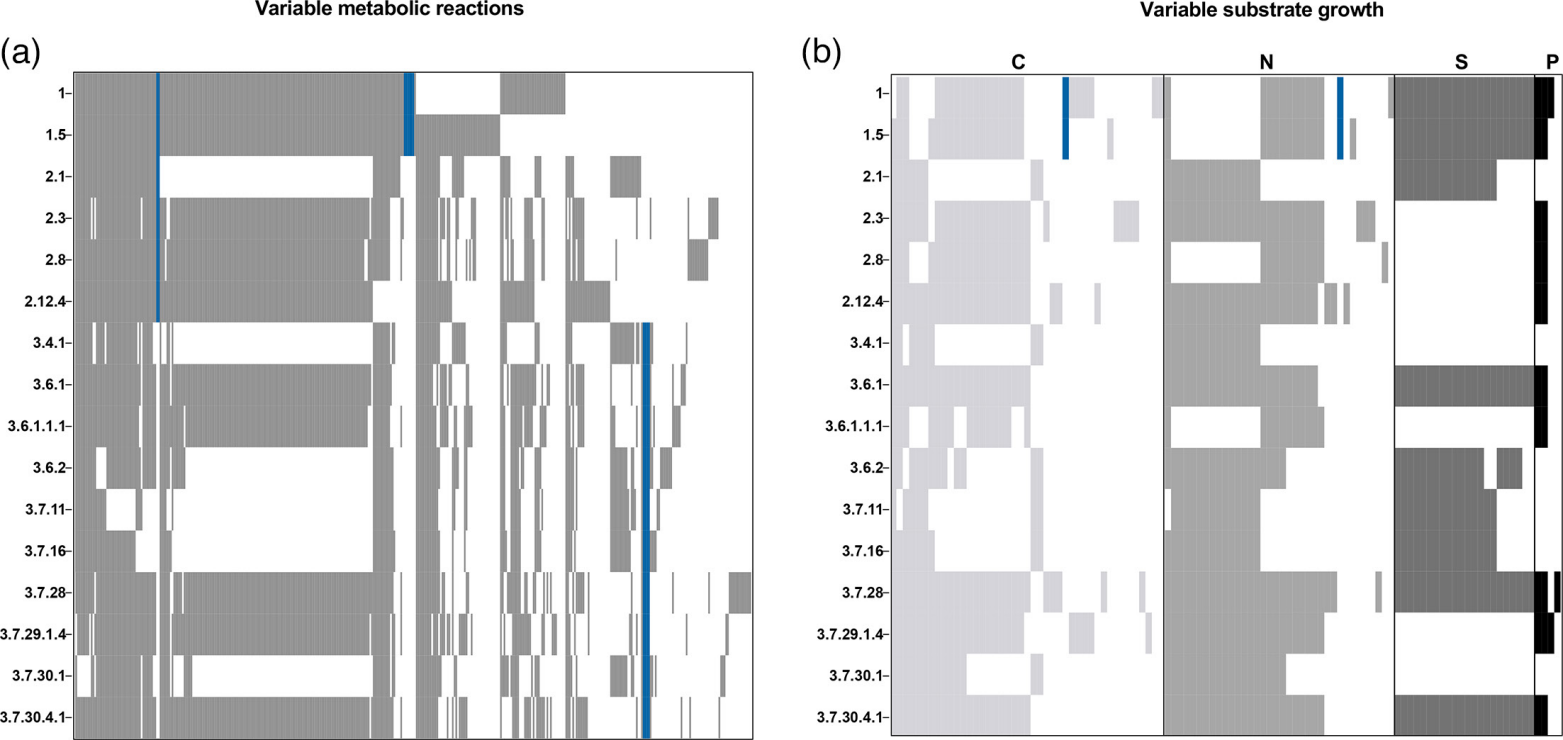
Heatmap of variable metabolic capacities predicted for each *S. sonnei* genome. (**a**) Presence or absence of variable metabolic reactions in each strain. Grey indicates presence, white indicates absence and blue indicates reactions unique to one or more lineages, *n*=394 reactions presented. (**b**) Predicted growth simulations on carbon (C), nitrogen (N), sulphur (S) or phosphorus (P) based metabolites. Grey or black indicates presence, white indicates absence and blue indicates reactions unique to one or more lineages, *n*=105 substrates presented. Full genome-scale metabolic models and tables summarizing all metabolic reactions and growth capacities can be downloaded from https://doi.org/10.6084/m9.figshare.28302986.

The convergent loss of metabolic function has been previously documented in all *Shigella* subgroups [10,67] and likely represents a key step towards host adaptation. To investigate any lineage-associated trends in the metabolic capacity of *S. sonnei*, strain-specific genome-scale metabolic models were built, and growth capacities were simulated. On average, the number of metabolic reactions predicted for each lineage was similar (Fig. 5a, Table S5); a total of 2,898 metabolic reactions were identified; of these, 2,505 were shared between all strains. A median of 2,748 reactions was identified in lineage 1 strains (range 2,742 to 2,753), 2741 in lineage 2 (range 2,625 to 2,746) and 2,691 in lineage 3 strains (range 2,602 to 2,749). Few reactions were specific to a single lineage: seven were predicted in only lineage 1 isolates (alpha amylase, periplasmic alpha-amylase, dextrin import, starch import, glutathione transport, succinate dehydrogenase and l-cysteine-specific tryptophanase); two predicted only in lineage 1 and 2 isolates (betaine-aldehyde dehydrogenase and a raffinose proton symporter) and four predicted in only lineage 3 isolates (a d-galactose ABC transporter which facilitates glutamate synthesis and a polysaccharide ABC transporter which imports maltotetraose, melibiose and raffinose). Intriguingly, the genes underlying these four reactions are truncated or disrupted in lineage 3 genomes, suggesting that these reactions may reflect annotation artefacts arising from pseudogenization rather than true metabolic differences, and their functional relevance will require experimental verification in future. For growth simulations, no growth was detected under anaerobic conditions on any substrates (despite *Shigella* being a facultative anaerobe), so only aerobic growth was considered here. Consistent with the metabolic reaction data, we identified a median of 410 predicted growth phenotypes in lineage 1 (range 408–412), 398 (range 384–407) in lineage 2 and 396 (range 374–422) in lineage 3. All strains were predicted to grow on 350 substrates, with variability observed for 105 substrates (Fig. 5b). The predicted ability to utilize l-tryptophan was restricted to lineage 1, which likely represents another instance of adaptive change, since the inability to utilize tryptophan (resulting in the indole-negative phenotype) has been observed in parallel across many *Shigella* lineages [68]. Otherwise, there were no predicted growth phenotypes that were unique to a single lineage. This variation between the presence of reactions and predicted growth could be explained by the exclusion of anaerobic growth conditions in our analysis, as well as being unable to model unknown/novel metabolism likely encoded within *Shigella*. Another limitation of this approach is the lack of phenotypic data available for *S. sonnei*, which left us unable to validate these models (although we note the similarity of * S. sonnei*’s metabolic capacity to *E. coli*, for which CarveMe models have demonstrated accuracy [49]). Overall, this data suggests a trend towards metabolic streamlining in lineage 3 *S*. *sonnei*; however, the differences observed here are minor and would benefit from functional verification.

## Conclusions

Here, we report the completed genomes of 15 *S*. *sonnei* isolates, representing epidemiologically relevant and phylogenetically distinct genotypes. The bacterial isolates and complete reference genomes made available here support future experimental and computational research on *S. sonnei*. For example, recent work utilizing this collection demonstrated significant variation in virulence phenotypes in both zebrafish and human neutrophil infection models, proving insights into factors driving the success of specific *S. sonnei* clones. These resources facilitate experimental studies that more accurately reflect contemporary infections and can be used to uncover mechanisms of pathogen success, informing vaccine and therapeutic development. Furthermore, the in-depth characterization of these complete genome sequences highlights ongoing adaptive evolution in *S. sonnei* and identifies areas deserving of further functional investigation to better understand *S. sonnei* biology, which remains vastly understudied in comparison to *S. flexneri.*

## Supplementary material

10.1099/mgen.0.001596 Uncited Supplementary Material 1.

## Abbreviations

AMR: antimicrobial resistance
HGC: homologous gene cluster
IS: insertion sequence
SNV: single nucleotide variant
SRL: *Shigella* resistance locus
