# Supplementary material for: A public resource of 15 genomically characterized representative strains of Shigella sonnei
Source: Microb Genom. 2026 Jan 12;12(1):001596. doi: 10.1099/mgen.0.001596 (PMC12795559; doi:10.1099/mgen.0.001596)
Supplement: Uncited Supplementary Material 1. [file mgen-12-01596-s001.pdf]

Figure S1 - Clinker gene cluster comparison of Tn7/Int2 and *Shigella* resistance loci variants.

A Tn7 / In2 insertions

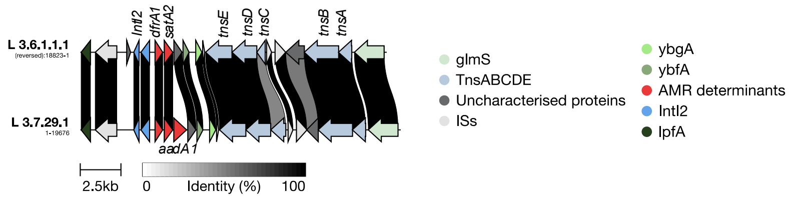

B SRL insertions

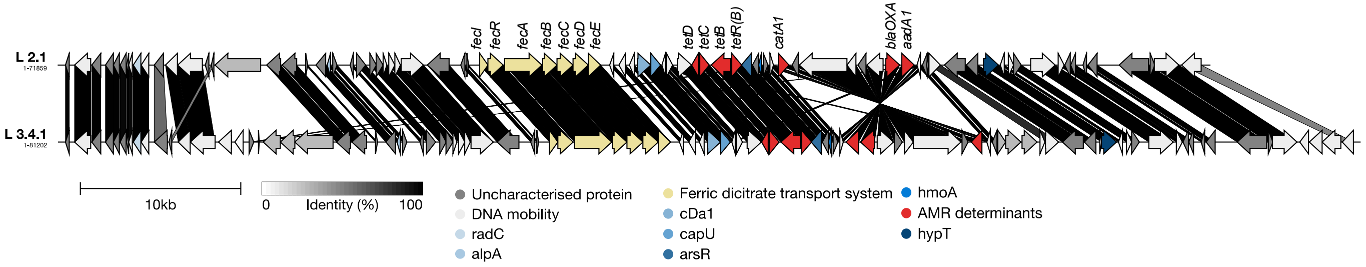

Figure S2 -Whole genome alignment of *S. sonnei* chromosomal sequences using progressiveMauve.

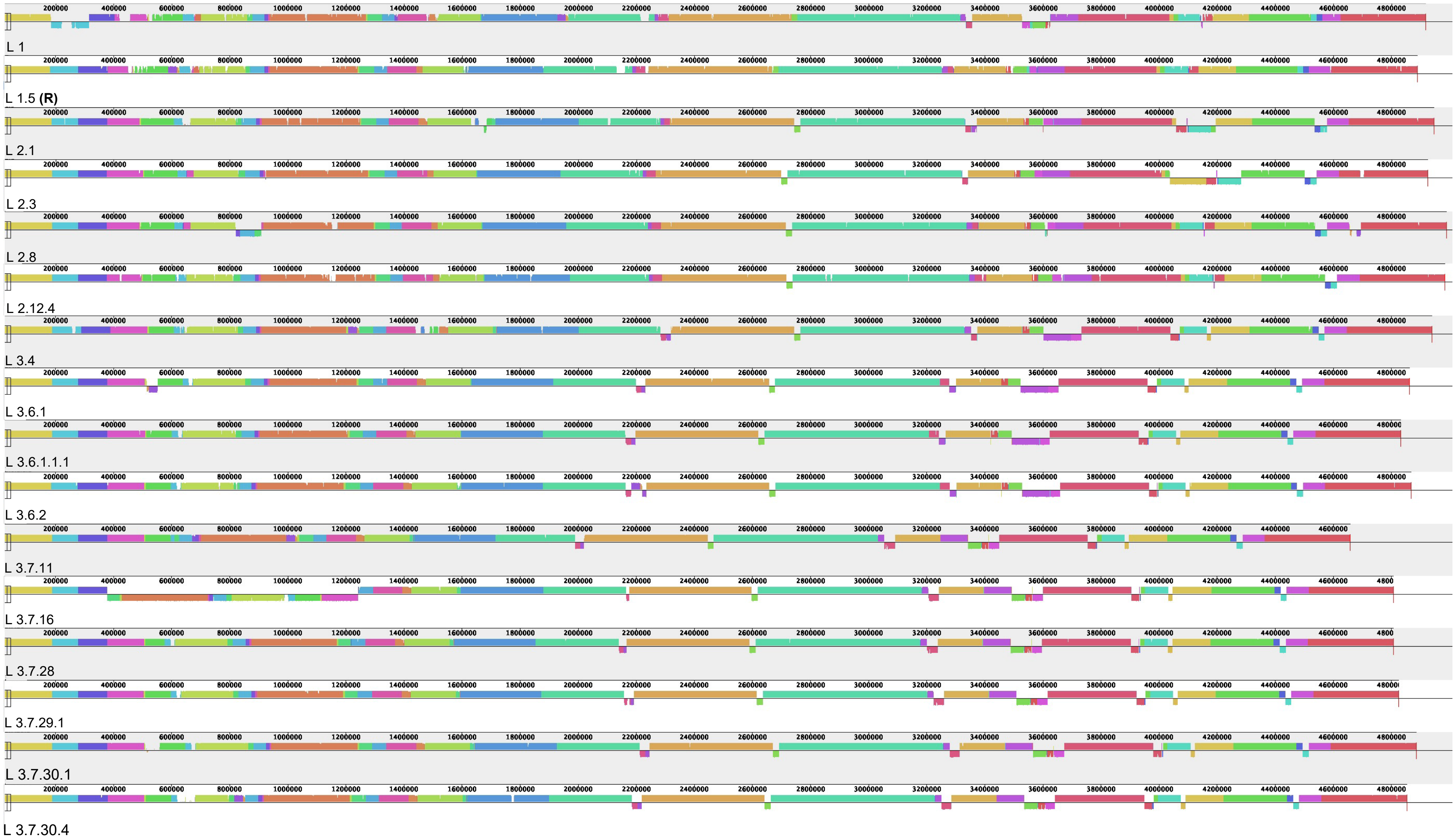

Figure S3 - Whole genome alignment of *S. sonnei* pINV sequences using progressiveMauve.

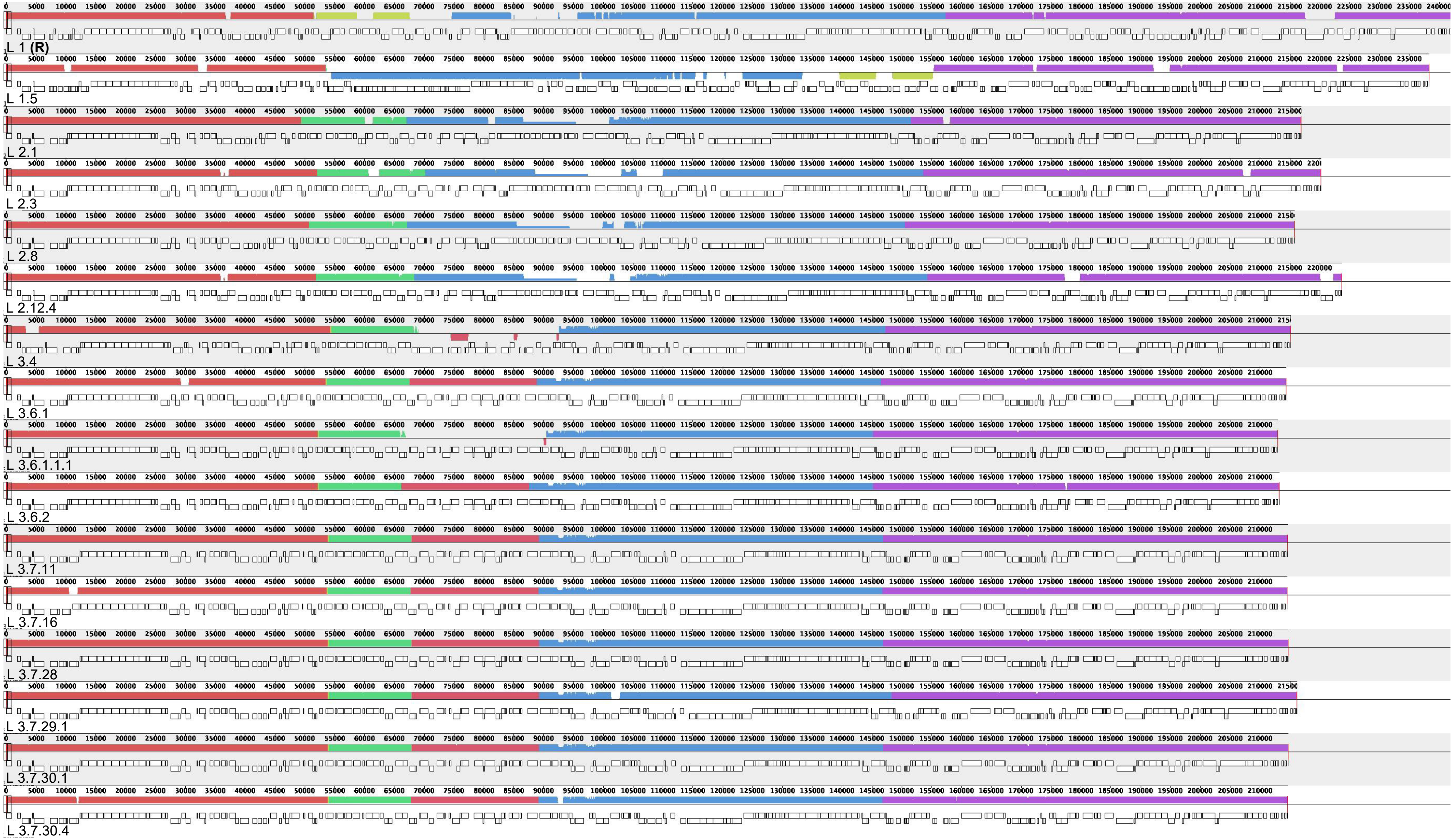

Table S1

Strain ID	Genotype[1]	Genome size (bp)	N50	GC (%)	ONT depth	CDSs[2]	tRNAs	rRNAs	Contigs	Chromosome size (bp)	pINV size (bp)	Other plasmids	Complete-ness[3]	Contami-nation[3]	Genbank accession	Culture collection accession	Year of isolation	Country of isolation	Citation
**201809330**	1	5389660	4916714	50.8	61x	5428	97	22	7	4916714	242731	5	99.54	0.26	CP180280-CP180286	CIP112510	2018	France	[21]
**391324**	1.5	5260824	4888493	50.9	59x	5255	97	22	4	4888493	238313	2	99.54	0.16	CP179997-CP180000	NCTC15123	2017	UK	N/A
**356538**	2.1	5170588	4946399	50.8	76x	5176	97	22	4	4946399	216858	2	99.56	0.11	CP179993-CP179996	NCTC15120	2017	UK	[14]
**830292**	2.3	5150027	4924717	50.8	60x	5166	96	22	3	4924717	220157	1	98.98	0.11	CP179990-CP179992	NCTC15131	2019	UK	[14]
**373220**	2.12.4	5289501	4983563	50.8	47x	5306	93	22	4	4983563	223662	2	99.6	0.11	CP179986-CP179989	NCTC15121	2017	UK	[14]
**590907**	3.4.1	5174681	4940164	50.8	74x	5229	97	22	7	4940164	215149	5	99.6	0.11	CP180015-CP180021	NCTC15124	2018	UK	[14]
**623218**	3.6.1	5112917	4862817	50.8	68x	5154	97	22	9	4862817	214316	7	99.6	0.26	CP179977-CP179985	NCTC15127	2018	UK	[14]
**02_1157**	3.6.1.1.1	5073758	4832017	50.8	86x	5110	96	22	5	4832017	212976	3	99.6	0.11	GCA_965230875.1	-	2014	Vietnam	[52]
**642321**	3.6.2	5100173	4866958	50.8	43x	5135	97	22	6	4866958	213229	4	99.6	0.11	CP179971-CP179976	NCTC15130	2018	UK	[14]
**633497**	3.7.11	4986415	4657310	50.8	46x	5046	93	22	7	4657310	214646	5	97.74	0.11	CP179964-CP179970	NCTC15129	2018	UK	[14]
**598955**	3.7.16	5039937	4807305	50.8	54x	5067	98	22	7	4807305	214536	5	99.6	0.11	CPI180008-CP180014	NCTC15125	2018	UK	[14]
**618335**	3.7.28	5138354	4806102	50.7	61x	5177	98	22	6	4806102	214666	4	99.6	0.58	CP179958-CP179963	NCTC15126	2018	UK	[14]
**03_0142**	3.7.29.1.4	5156461	4824935	50.8	86x	5182	96	22	9	4824935	214579	7	99.6	0.11	GCA_965230885.1	-	2014	Vietnam	[65]
**627346**	3.7.30.1	5122274	4884953	50.8	42x	5168	100	22	6	4884953	216152	4	99.6	0.11	CP179952-CP179957	NCTC15128	2018	UK	[14]
**381259**	3.7.30.4.1	5260132	4933886	50.7	58x	5322	91	22	8	4933886	214700	6	99.6	0.11	CP176607-CP176614	NCTC15122	2017	UK	[14]
**53G***	2.8	5220473	4988504	50.7	NA	5248	96	22	5	4988504	215774	3	NA	NA	HE616528-HE616532	-	1954	Japan	[11]

Table S2.

**Genotype**	**IncFIA/ IncFIC**	**MOB_F/ Col-E1-like**	**Col(BS512)**	**Col156/ MOB_Q**	**Col(MG828)**	**IncI2/ MOB_P**	**IncFIA,IncFII/ MOB_F,MOB_P**	**IncK2/Z/ MOB_P**	**IncI1/B/O /MOB_P**	**IncFIB**	**IncX1/MOB_P**	**IncI-gamma /K1/MOB_P**	**MOB^P**	**MOB_Q**	**No-MOB**
**1**	242731 bp*<br>***HE616529***	-	-	-	-	65348 bp*<br>***CP028155***<br>43917 bp*<br>***LT985310***	-	-	102906 bp*<br>***CP041565***	-	-	-	6888 bp<br>***CP014198***	11156 bp<br>***CP023649***	-
**1.5**	238313 bp<br>***CP000039***	-	-	-	-	-	125562 bp<br>***CP001065***	-	-	-	-	-	-	7084 bp<br>***CP023649***	-
**2.1**	216858 bp<br>***HE616529***	-	-	-	-	-	-	-	-	-	-	-	-	4074 bp<br>***CP018208***	3257 bp<br>***CP019023***
**2.3**	220157 bp<br>***HE616529***	-	-	-	-	-	-	-	-	-	-	-	5153 bp<br>***HE616530***	-	-
**2.8**	215774 bp<br>***HE616529***	5153 bp<br>***CP019897***	2089 bp<br>***HE616531***	-	-	-	-	-	-	-	-	-	-	-	8953 bp<br>***HE616532***
**2.12.4**	223662 bp<br>***CP023646***	-	-	-	-	-	77123 bp*<br>***AP014877***	-	-	-	-	-	5153 bp<br>***HE616530***	-	-
**3.4.1**	215149 bp<br>***CP023646***	-	2088 bp<br>***CP033398***	5114 bp<br>***CP019693***	-	-	-	-	-	-	-	-	2717 bp<br>***CP038000***	6750 bp<br>***DQ916145***	2699 bp*<br>***CP039609***
**3.6.1**	214316 bp<br>***CP023646***	2690 bp<br>***CP038000***	-	6015 bp<br>***KP970685***<br>5114 bp<br>***CP019693***	1549 bp<br>***CP003038***	-	-	-	-	-	-	-	7939 bp<br>***KU932034***	4076 bp<br>***CP011140***	8401 bp*<br>***CP034068***
**3.6.1.1.1**	212976 bp<br>***CP023646***	-	2089 bp<br>***CP030115***	-	-	-	-	-	-	-	-	-	2690 bp<br>***CP038000***	4878 bp<br>***KP970685***<br>4269 bp<br>***CP019693***	8401 bp*<br>***CP034068***<br>3787 bp<br>***CP019138***<br>2651 bp<br>***KX618698***
**3.6.2**	213229 bp<br>***CP023646***	2690 bp<br>***CP038000***	2089 bp<br>***CP030115***	5114 bp<br>***CP019693***	-	-	-	-	-	-	-	-	-	-	10093 bp*<br>***CP034068***
**3.7.11**	214646 bp<br>***CP016533***	2690 bp<br>***CP038000***	2101 bp<br>***CP023648***	5114 bp<br>***CP019693***	-	-	-	-	-	-	-	97999 bp<br>***CP016533***	6555 bp<br>***NC_008488***	-	-
**3.7.16**	214536 bp<br>***CP023646***	2690 bp<br>***CP038000***	2089 bp<br>***CP023648***	5114 bp<br>***CP019693***	1459 bp<br>***CP023264***	-	-	-	-	-	-	-	-	6744 bp<br>***KY348421***	-
**3.7.28**	214666 bp<br>***CP023646***	5153 bp<br>***CP019897***<br>2690 bp<br>***CP019897***	-	-	-	-	-	-	-	108503<br>bp<br>***CP03406<br>6***	-	-	1240 bp<br>***CP018984***	-	-
**3.7.29.1.4**	214579 bp<br>***CP023646***	2690 bp<br>***CP023653***<br>-	2101 bp<br>***CP023648***	-	1549 bp<br>***CP003038***	-	-	96229 bp<br>***CP026855***	-	-	-	-	-	6774 bp<br>***NC_022585***	4869 bp<br>***CP030008***<br>2735 bp<br>***NC_011405***
**3.7.30.1**	216152 bp<br>***CP023646***	5153 bp<br>***CP023650***<br>2690 bp<br>***CP038000***	-	-	-	-	-	-	-	-	-	-	-	-	8212 bp<br>***CP025235***
**3.7.30.4.1**	214700 bp<br>***CP023646***	7717 bp<br>***CP023650***	2101 bp<br>***CP023648***	5114 bp<br>***CP019693***	-	-	-	57356 bp<br>***CP018984***	-	-	35184 bp<br>***CP002111***	-	-	4074 bp<br>***LT985255***	-

Table S3

Genotype	IncFIA/ IncFIC	MOB_F/Col-E1-like	Col(BS512)	Col156/ MOB_Q	Col(MG828)	IncI2/ MOB_P	IncFIA,IncFII/ MOB_F,MOB_P	IncK2/Z /MOB_P	IncI1/B/O /MOB_P	IncFIB	IncX1/ MOB_P	IncI-gamma/K1 / MOB_P	MOB_P	MOB_Q	No-MOB
**1**	-	-	-	-	-	- -	-	-	-	-	-	-	*cea* (Colicin-E1)	*ceaC* (Colicin-E3)	-
**1.5**	-	-	-	-	-	-	-	-	-	-	-	-	-	-	-
**2.1**	-	-	-	-	-	-	-	-	-	-	-	-	-	*ceaC* (Colicin-E3)	-
**2.3**	-	-	-	-	-	-	-	-	-	-	-	-	-	*ceaC* (Colicin-E3)	-
**2.8**	-	*cea* (Colicin-E1)	-	-	-	-	-	-	-	-	-	-	-	-	-
**2.12.4**	-	-	-	-	-	-		-	-	-	-	-	-	*ceaC* (Colicin-E3)	-
**3.4.1**	-	-	-	-	-	-	-	-	-	-	-	-	-	*col* (Colicin-E9)	-
**3.6.1**	-	-	-	(Colicin-E3) -	-	-	-	-	-	-	-	-	-	-	-
**3.6.1.1.1**	-	-	-	-	-	-	-	-	-	-	-	-	-	(Colicin-E3) -	- - -
**3.6.2**	-	-	-	-	-	-	-	-	-	-	-	-	-	-	-
**3.7.11**	-	-	-	-	-	-	-	-	-	-	-	*cia* (Colicin Ia)	-	-	-
**3.7.16**	-	-	-	-	-	-	-	-	-	-	-	-	-	*col* (Colicin-E9)	-
**3.7.28**	-	*cea* (Colicin-E1) -	-	-	-	-	-	-	-	-	-	-	-	-	-
**3.7.29.1.4**	-	- -	-	-	-	-	-	-	-	-	-	-	-	(Colicin-E5)	- -
**3.7.30.1**	-	*cea* (Colicin-E1) -	-	-	-	-	-	-	-	-	-	-	-	-	-
**3.7.30.4.1**	-	(Colicin 10)	-	-	-	-	-	-	-	-	-	-	-	-	-

Table S4

Genotype	IncFIA/IncFIC	MOB$_F$/Col-E1-like	Col(BS512)	Col156/MOB$_Q$	Col(MG828)	IncI2/MO BP	IncFIA,IncFII/ MOB$_F$,MO BP	IncK2/Z /MO BP	IncI1/B/O /MO BP	IncFIB	IncX1/MO BP	IncI-gamma/K1 /MO BP	MO BP	MOB$_Q$	No-MOB
**1**	*blaTEM-1*	-	-	-	-	*blaTEM-1*<br>*blaTEM-1*	-	-	*aph(6)-Id*<br>*blaTEM-1*<br>*dfrA14*<br>*aph(3'')-Ib*<br>*sul2*	-	-	-	-	-	-
**1.5**	-	-	-	-	-	-	-	-	-	-	-	-	-	-	-
**2.1**	-	-	-	-	-	-	-	-	-	-	-	-	-	-	-
**2.3**	-	-	-	-	-	-	-	-	-	-	-	-	-	-	-
**2.8**	-	-	-	-	-	-	-	-	-	-	-	-	-	-	-
**2.12.4**	-	-	-	-	-	-	*aph(6)-Id*<br>*blaTEM-1*<br>*aph(3'')-Ib*<br>*sul2*<br>*tet(B)*	-	-	-	-	-	-	-	-
**3.4.1**	-	-	-	-	-	-	-	-	-	-	-	-	-	-	*qnrB19*
**3.6.1**	-	-	-	-<br>-	-	-	-	-	-	-	-	-	-	-	*aph(6)-Id*<br>*aph(3'')-Ib*<br>*sul2*<br>*tet(A)*
**3.6.1.1.1**	-	-	-	-	-	-	-	-	-	-	-	-	-	-<br>-	*aph(6)-Id*<br>*aph(3'')-Ib*<br>*sul2*<br>*tet(A)*<br>-<br>-
**3.6.2**	-	-	-	-	-	-	-	-	-	-	-	-	-	-	*aph(6)-Id*<br>*aph(3'')-Ib*<br>*sul2*<br>*tet(A)*
**3.7.11**	-	-	-	-	-	-	-	-	-	-	-	-	-	-	-
**3.7.16**	-	-	-	-	-	-	-	-	-	-	-	-	-	-	-
**3.7.28**	-	-<br>-	-	-	-	-	-	-	-	*dfrA51*	-	-	-	-	-
**3.7.29.1.2**	-	-<br>-	-	-	-	-	-	*aph(6)-Id*<br>*aph(3'')-Ib*<br>*sul2*	-	-	-	-	-	-	-<br>-
**3.7.30.1**	-	-	-	-	-	-	-	-	-	-	-	-	-	-	-
**3.7.30.4.1**	-	-	-	-	-	-	-	-	-	-	-	-	-	-	-

Supplementary material

Gene list of structural variants too large to be inserted into Table 5.

~ 130 kbp inversion in clades 3.4 and 3.6

Hypothetical protein, *yhiM, yhiN, pitA, uspB, uspA, dtpB, rsmJ, prlC, rlmJ, gorA,* hypothetical protein, *arsR, arsB, arsC, arsR,* hypothetical protein, *tnp, istB,* hypothetical protein, *tnp, tnp, tnp, tnp, yhiS*, hypothetical protein, *slp , dctR, yhiD, hdeB, hdeA, hdeD, gadE,* hypothetical protein, *mdtE, mdtF,* hypothetical protein, *gadW, gadX, gadB, ccp , treF, tnp, yieL,* hypothetical protein, hypothetical protein, *cbrC,* hypothetical protein, hypothetical protein, *cbrB, yieH, adeP, chrR,* hypothetical protein, *yieE, yidZ, mdtL*

~107 kbp translocation inversion in clade 3.7

mnmE, yidC, rnpA, rpmH, ysdD, dnaA, dnaN, recF, gyrB, yidB, yidA, yidX, dgoR, dgoK, dgoA, dgoD, dgoD, dgoT, cbrA, yidR, yidQ, ibpA, ibpB, yidE, yidP, glvC, hypothetical protein, *celF, insQ, yidI, yidH, yidG, yidF, emrD, ysdE, tisB,* hypothetical protein, *ilvN, uhpA, uhpB, uhpC, uhpT, adeD, tnp*, hypothetical protein, *yicN, nepI, yicS, nlpA,* hypothetical protein, hypothetical protein, hypothetical protein, hypothetical protein, tnp, hypothetical protein, tnp, *tnp,* hypothetical protein, *tnp, iutA, iucD, iucC, iucB, iucA, shiF,* hypothetical protein, hypothetical protein, *tnp, tnp, tnp,* hypothetical protein, *tra5,* hypothetical protein, *tnp, tnp,* hypothetical protein, *tnp, istB,* hypothetical protein, *tnp, tnp, tnp, tnp, yhiS,* hypothetical protein, *slp , dctR, yhiD, hdeB, hdeA, hdeD, gadE,* hypothetical protein, *mdtE, mdtF,* hypothetical protein, *gadW, gadX, gadB, ccp , treF,* hypothetical protein, *tnp, yieL,* hypothetical protein, *tnp, cbrC,* hypothetical protein, hypothetical protein, *cbrB, yieH, adeP, chrR,* hypothetical protein, *yieE, yidZ, mdtL*
